# Generation of disease-specific induced pluripotent stem cells from patients with rheumatoid arthritis and osteoarthritis

**DOI:** 10.1186/ar4470

**Published:** 2014-02-04

**Authors:** Jaecheol Lee, Youngkyun Kim, Hyoju Yi, Sebastian Diecke, Juryun Kim, Hyerin Jung, Yeri Alice Rim, Seung Min Jung, Myungshin Kim, Yong Goo Kim, Sung-Hwan Park, Ho-Youn Kim, Ji Hyeon Ju

**Affiliations:** 1 Division of Cardiology, Department of Medicine, Stanford University School of Medicine, 265 Campus Drive, Room G1120B, Stanford, CA 94305-5454, USA; 2 Institute for Stem Cell Biology and Regenerative Medicine, Stanford University School of Medicine, 265 Campus Drive, Room G1120B, Stanford, CA 94305-5454, USA; 3 Stanford Cardiovascular Institute, Stanford University School of Medicine, 265 Campus Drive, Room G1120B, Stanford, CA 94305-5454, USA; 4 Division of Rheumatology, Department of Internal Medicine, Seoul St. Mary’s Hospital, Institute of Medical Science, College of Medicine, The Catholic University of Korea, #505, Banpo-Dong, Seocho-Gu, Seoul 137-701, South Korea; 5 Department of Laboratory Medicine, College of Medicine, Catholic Genetic Laboratory Center, Seoul St. Mary’s Hospital, The Catholic University of Korea, #505, Banpo-Dong, Seocho-Gu, Seoul 137-701, South Korea

## Abstract

**Introduction:**

Since the concept of reprogramming mature somatic cells to generate induced pluripotent stem cells (iPSCs) was demonstrated in 2006, iPSCs have become a potential substitute for embryonic stem cells (ESCs) given their pluripotency and “stemness” characteristics, which resemble those of ESCs. We investigated to reprogram fibroblast-like synoviocytes (FLSs) from patients with rheumatoid arthritis (RA) and osteoarthritis (OA) to generate iPSCs using a 4-in-1 lentiviral vector system.

**Methods:**

A 4-in-1 lentiviral vector containing Oct4, Sox2, Klf4, and c-Myc was transduced into RA and OA FLSs isolated from the synovia of two RA patients and two OA patients. Immunohistochemical staining and real-time PCR studies were performed to demonstrate the pluripotency of iPSCs. Chromosomal abnormalities were determined based on the karyotype. SCID-beige mice were injected with iPSCs and sacrificed to test for teratoma formation.

**Results:**

After 14 days of transduction using the 4-in-1 lentiviral vector, RA FLSs and OA FLSs were transformed into spherical shapes that resembled embryonic stem cell colonies. Colonies were picked and cultivated on matrigel plates to produce iPSC lines. Real-time PCR of RA and OA iPSCs detected positive markers of pluripotency. Immunohistochemical staining tests with Nanog, Oct4, Sox2, Tra-1-80, Tra-1-60, and SSEA-4 were also positive. Teratomas that comprised three compartments of ectoderm, mesoderm, and endoderm were formed at the injection sites of iPSCs. Established iPSCs were shown to be compatible by karyotyping. Finally, we confirmed that the patient-derived iPSCs were able to differentiate into osteoblast, which was shown by an osteoimage mineralization assay.

**Conclusion:**

FLSs derived from RA and OA could be cell resources for iPSC reprogramming. Disease- and patient-specific iPSCs have the potential to be applied in clinical settings as source materials for molecular diagnosis and regenerative therapy.

## Introduction

The concept of reprogramming mature somatic cells to generate induced pluripotent stem cells (iPSCs) was demonstrated by Takahashi and Yamanaka in 2006 [1]. Four factors, namely Oct4, Klf4, Sox2, and c-Myc, were transduced into somatic cells to reprogram and generate iPSCs. Subsequently, iPSCs have become a potential substitute for embryonic stem cells (ESCs) given their pluripotency and stemness characteristics, which resemble those of ESCs [2,3]. iPSCs may have important potential clinical applications as drug screening platforms, in pathophysiological studies in dishes, and as candidate cell sources for regenerative medicine [4-7].

The iPSCs used in pathophysiological studies in dishes were generated from the primary cells that originated from patients with neurological, hematological, metabolic, cardiovascular, primary immunodeficiency diseases, and so on [5,8-10]. These pioneering studies have detected many novel pathophysiological mechanisms, which were impossible to study previously because of the inaccessibility of disease tissues and cells. Patient-specific iPSCs are particularly useful for studying diseases with complex mechanisms, which are affected by several factors that range from the genetic background to environmental modifications.

Rheumatoid arthritis (RA) may be a promising target disease for iPSC applications because of its complex pathophysiology. The iPSCs from RA patients could be extended to a regenerative approach via their differentiation into mature chondrocytes and osteocytes, which synthesize cartilage and bone. We therefore selected fibroblast-like synoviocytes (FLSs) from RA and osteoarthritis (OA) for reprogramming using a four-in-one lentiviral vector, which contained four factors: Oct4, Klf4, Sox2, and c-Myc. RA FLSs, which are regarded as major pathophysiological players in RA, are thought to be a good candidate for reprogramming to simulate the disease RA in dishes [11-13].

In this study, we successfully reprogrammed RA FLSs and OA FLSs to generate disease-specific iPSCs. Their pluripotency was demonstrated by immunohistochemical staining and teratoma formation *in vivo*. To our knowledge, this is the first attempt to induce stem cells from RA FLSs.

## Methods

### Patient recruitment and synovia preparation

Four patients were used for synovia preparation. RA patients (*n* = 2) who satisfied the 1987 revised criteria of the American College of Rheumatology (formerly the American Rheumatism Association) [14] and OA patients (*n* = 2) were recruited from the outpatient clinic at the Department of Rheumatology, Seoul St. Mary’s Hospital, Seoul, South Korea. These patients had received arthroscopic synovectomy or total knee replacement surgery. Synovia samples were obtained during the operations. Eligibility for OA inclusion required that individuals had primary knee OA, which was diagnosed according to American College of Rheumatology criteria. The experimental protocol was approved by the Catholic University of Korea Human Research Ethics Committee.

### Isolation and maintenance of RA and OA fibroblast-like synoviocytes

RA and OA synovia were stored in the sample bank of the Rheumatism Research Center, Seoul, South Korea. These samples were classified only by disease and stored in a blind manner. The institutional review board permitted that a patient consent form was not necessary because samples were anonymous and did not contain patient information. Tissues were homogenized and resuspended in Dulbecco’s modified Eagle’s medium (DMEM) (Gibco by Invitrogen, Carlsbad, CA, USA) containing 0.01% collagenase and were incubated for 4 hours at 37°C with vigorous shaking. Cells were washed and resuspended in DMEM supplemented with 20% fetal bovine serum (FBS) (Gibco by Invitrogen), and 1% penicillin/streptomycin solution (Gibco by Invitrogen). Cells were cultured until the adherent fibroblast cells achieved confluence.

### Lentivirus production and transduction

293FT cells (Invitrogen, Carlsbad, CA, USA) were plated at 80% confluence in 100-mm dishes and transfected with 12 μg of four-in-one reprogramming plasmid (Oct4, Sox2, Klf4, and c-Myc), 9 μg packaging pPAX2 plasmids, and 3 μg pMD2G plasmids using Lipofectamine 2000 (Invitrogen). After about 48 to 72 hours of culture, the virus was harvested and mixed with Lenti-X Concentrator (Clontech Laboratories, Mountain View, CA, USA). After overnight incubation at 4°C, viruses were precipitated at 1,500 × *g* and resuspended in phosphate-buffered saline. For virus infection, RA or OA FLSs were seeded onto six-well plates. The lentivirus was applied with culture medium for overnight infection. The iPSC colonies were picked after 18 to 20 days of reprogramming.

### Cell culture and maintenance of patient-specific iPSCs

RA or OA FLSs were maintained in DMEM containing 20% FBS at 37°C, with 95% air and 5% CO_2_ in a humidified incubator. All of the cells used for reprogramming were at passage 8. Patient-specific iPSCs were maintained in Matrigel-coated tissue culture dishes (BD Biosciences, San Jose, CA, USA) with E8 human ESC medium.

### Quantitative real-time polymerase chain reaction

Total RNA was isolated using an RNeasy Plus Mini Kit (Qiagen, Valencia, CA, USA). Reverse transcriptase polymerase chain reaction was performed using an iScript™ cDNA Synthesis Kit (BIORAD, Marnes-La-Coquette, France). Gene expression was quantified by SYBR Green real-time polymerase chain reaction using an ABI Prism 7300 Sequence Detection System (Applied Biosystems, Foster City, CA, USA). The relative mRNA levels were normalized to the values of GAPDH mRNA for each reaction. The primer sequences are described in Additional file 1.

### Immunostaining

The iPSC clones were fixed with 4% paraformaldehyde and immunostaining was performed using the following primary antibodies: SSEA-4, Tra-1-60 and Tra-1-80 (Millipore, Billerica, MA, USA), Oct3/4 and Nanog (Santa Cruz Biotechnology, Santa Cruz, CA, USA), and Sox2 (BioLegend, San Diego, CA, USA). Samples were incubated with Alexa Fluor 594-conjugates or 488-conjugated secondary antibody (Invitrogen) and detected by indirect immunofluorescence microscopy.

### Teratoma formation

Teratoma formation was performed and analyzed with the approval of the Institutional Animal Care and Use Committee of Applied StemCell (protocol number APP-12-001-Y2; Sunnyvale, CA, USA). Briefly, undifferentiated iPSCs (1 × 10^6^) were suspended in 10 μl Matrigel (BD Biosciences) and delivered using a 28.5 gauge syringe into the subrenal capsule of 8-week-old SCID-beige mice. Eight weeks after cell delivery, the tumors were explanted and subjected to hematoxylin and eosin staining.

### Osteogenic differentiation

For osteogenic differentiation, we cultured iPSCs in osteogenic differentiation medium (ODM) as described by Kao and colleagues [15]. ODM is DMEM supplemented with 15% FBS, 50 μg/ml ascorbate-2-phosphate, 10 nmol/l dexamethasone, and 10 mmol/l β-glycerophosphate. At day 7 after osteogenic induction, *in vitro* mineralization of cells was assessed using the OsteoImage Mineralization Assay kit (Lonza, Basel, Switzerland) according to the manufacturer’s manual. Fluorescent signals from the hydroxyapatate portion were detected by fluorescence microscope (Axio observer 2.1; ZEISS, Thornwood, NY 10594, USA).

### Karyotyping

We added 30 μl Chromosome Resolution Additive (Genial Genetic Solutions Ltd, Runcorn, UK) to each six-well plate. After 1 hour of incubation, colcemid® was treated for 30 minutes. Cells were harvested using trypsin and treated by prewarmed hypotonic solution (KCl). Fixation was then performed with 1:3 acetic acid:methanol solution and the slide was prepared for chromosome analysis. The chromosome analysis was performed using a trypsin-Giemsa banding technique. At least 20 metaphases were analyzed.

### Ethics statement

This study protocol was approved by the institutional review board of The Catholic University of Korea (KC12TISI0861).

## Results

### Isolation of RA and OA fibroblast-like synoviocytes and successful generation of disease-specific iPSCs

**Figure 1:**
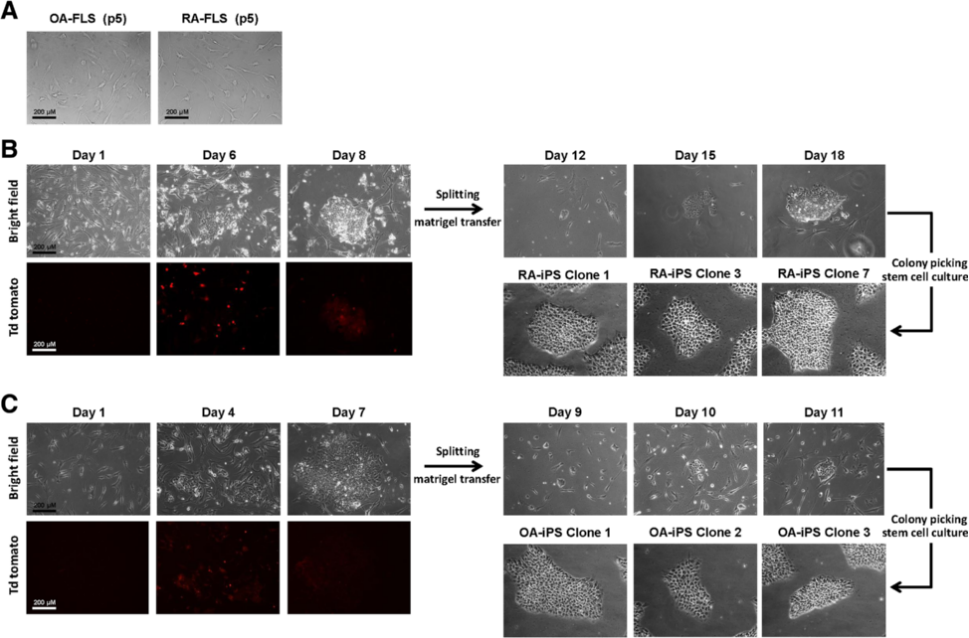
**Successful generation of induced pluripotent stem cells from rheumatoid arthritis and osteoarthritis fibroblast-like synoviocytes. (A)** Rheumatoid arthritis (RA) fibroblast-like synoviocytes (FLSs) and osteoarthritis (OA) FLSs were cultured before reprogramming. RA and OA FLS was extracted from RA patients (*n* = 2) and OA patients (*n* = 2) and were cultured as previously documented. There were five passages of RA and OA FLSs. Synovium was gathered from the elbow and knee during synovectomy surgery. One sample (RA1) was from a disease-modifying antirheumatic drug-naïve patient and the other sample (RA2) from a biologics-refractory patient with persistent high disease activity. **(B)** Tomato fluorescence was shown shortly after virus infection. On the 6th day after the transduction of four-in-one viral vector on RA FLSs, colony-like spheres began to form. On the 18th day, colonies were picked and resuspended on Matrigel-coated culture dishes. **(C)** Tomato fluorescence was positively shown on four-in-one vector-transduced OA FLSs. On the 7th day after the transduction of four-in-one viral vector on OA FLS, colony-like spheres began to form. On the 11th day, colonies were picked and resuspended on Matrigel-coated culture dishes.

We cultured FLSs from patients with RA (*n* = 2) and with OA (*n* = 2) (Figure 1A). The four-in-one vector was transduced into RA FLSs and OA FLSs, and they were serially observed. Colonies were formed and picked manually after 18 to 20 days. They were cultured through five passages (Figure 1B and 1C).

### Positive expression of pluripotency markers by RA and OA iPSCs

**Figure 2:**
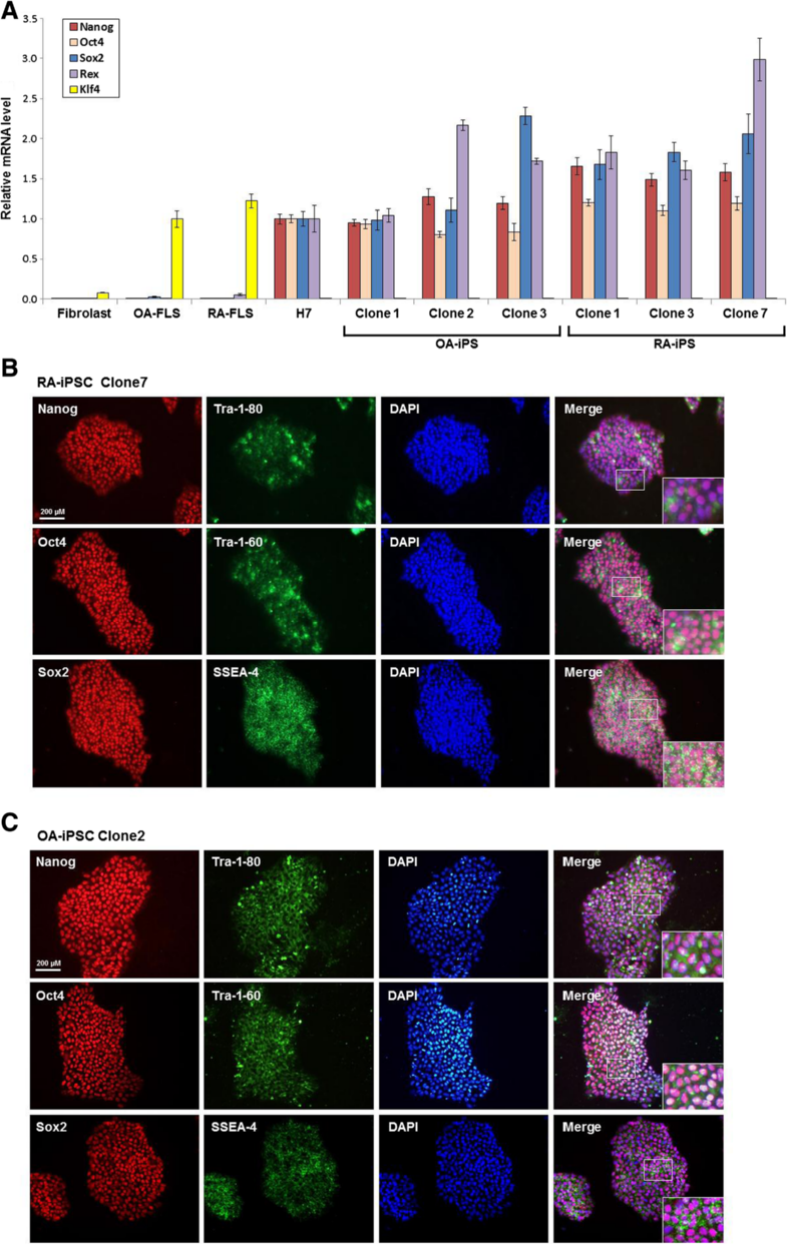
**Positive expression of pluripotency marker on osteoarthritis and rheumatoid arthritis induced pluripotent stem cells. (A)** The expression of stemness genes in osteoarthritis (OA) induced pluripotent stem cells (iPSCs) and rheumatoid arthritis (RA) iPSCs was analyzed by quantitative reverse transcriptase polymerase chain reaction (RT-PCR) analysis. Total RNA of fibroblast, OA fibroblast-like synoviocytes (FLSs), RA FLSs, H7, OA iPSCs and RA iPSCs was isolated and analyzed for endogenous pluripotent genes by RT-PCR analysis. The expression of endogenous Nanog, Oct4, Sox2 and Rex is upregulated in OA iPSCs and RA iPSCs. OA FLS and RA FLS already expressed Klf4 before reprogramming process started. **(B)** Immunofluorescence staining against Nanog, Oct4, Sox2, Tra-1-80, Tra-1-60, and SSEA-4 in RA iPSCs. RA iPSCs expressed a high level of these markers. **(C)** Immunofluorescence staining against Nanog, Oct4, Sox2, Tra-1-80, Tra-1-60, and SSEA-4 in OA iPSCs. OA iPSCs were positive for the pluripotency genes such as Nanog, Oct4, Sox2, Tra-1-80, Tra-1-60, and SSEA-4.

RA and OA iPSCs were immunostained against Nanog, Oct4, Sox2, Tra-1-80, Tra-1-60, and SSEA-4. These factors all stained positively in six iPSC clones that originated from two RA patients and two OA patients (Figure 2B, 2C and Additional file 2). The pluripotency mRNA markers for Nanog, Oct4, Sox2, Klf4, and Rex also increased, according to the real-time reverse transcriptase polymerase chain reaction analysis (Figure 2A). Interestingly, the level of Klf4 was elevated before reprogramming in RA FLSs and OA FLSs. Other factors began to increase after reprogramming and they peaked at higher levels than those found in H7 cells.

### Successful teratoma formation and normal karyotyping

**Figure 3:**
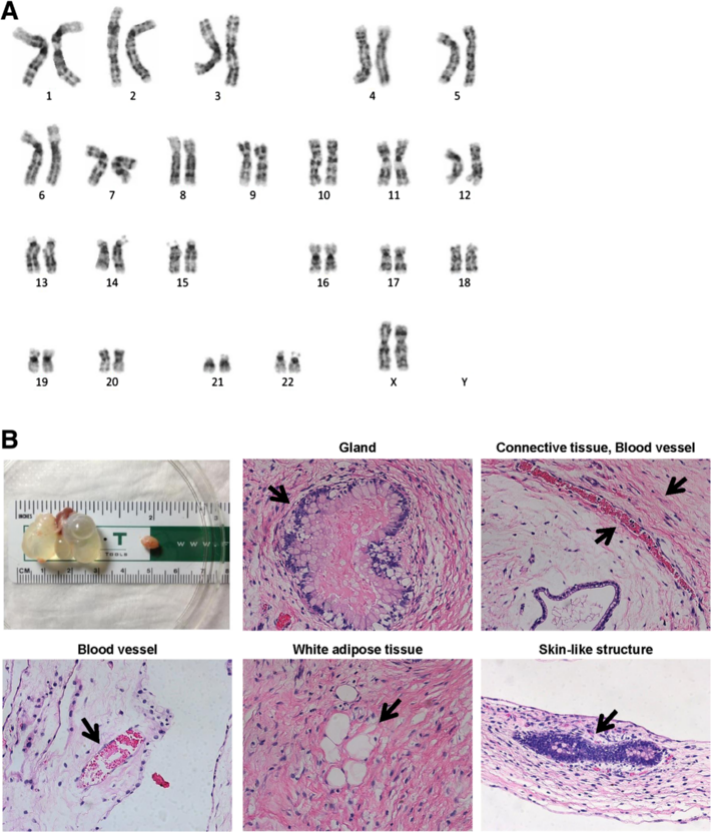
**Characterization of rheumatoid arthritis induced pluripotent stem cells. (A)** High-resolution, G-banded karyotype indicated a normal diploid female chromosomal content 46XX in rheumatoid arthritis (RA) induced pluripotent stem cells (iPSCs). **(B)** Teratoma formation occurred after injection of RA iPSCs into immunocompromised mice. Tumors were detected from the sites of injection and harvested after 3 months, and were examined for the presence of cells of three embryonic germ layers. Teratomas contained tissue from all three germ layers, including the gland (endoderm), blood vessel (mesoderm), adipose tissue (mesoderm), and skin-like structure (ectoderm).

The RA iPSC karyotype had a normal chromosomal pattern of 44 + XX (Figure 3A). RA iPSCs were injected into the kidney capsule and testis of SCID mice. At 12 weeks, teratomas formed successfully and they comprised three compartments: ectoderm, mesoderm, and endoderm (Figure 3B). Each compartment contained glandular tissue, connective tissue, blood vessels, adipose tissue, and skin-like structures.

### Osteogenic differentiation of RA and OA iPSCs

**Figure 4:**
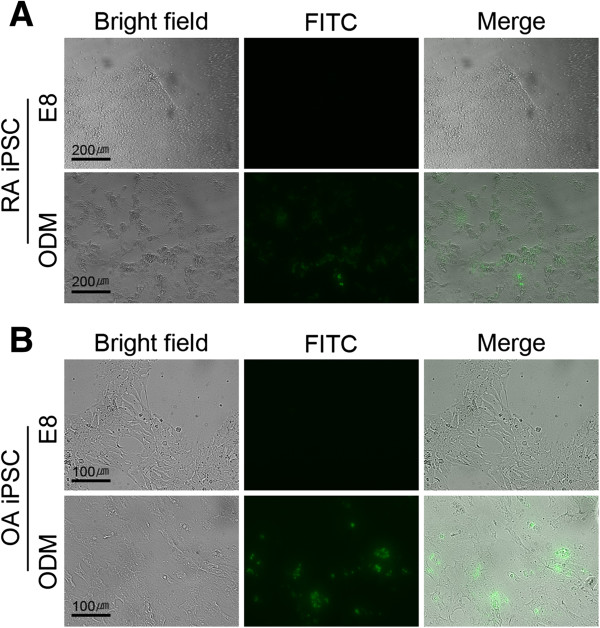
***In vitro *osteogenic differentiation of rheumatoid arthritis and osteoarthritis induced pluripotent stem cells.** To induce osteogenic differentiation, rheumatoid arthritis (RA) and osteoarthritis (OA) induced pluripotent stem cells (iPSCs) were cultured in E8 medium or osteogenic differentiation medium (ODM) for 7 days. Osteogenic differentiation was determined by the fluorescence detection for mineralization. Osteogenic mineralization was detected in both ODM-treated RA iPSCs **(A)** and OA iPSCs **(B)** by fluorescence microscope (fluorescein isothiocyanate (FITC)).

To confirm the osteogenic capability of patient-derived iPSCs, we tried to differentiate RA and OA patient-specific iPSCs to osteocytes. RA iPSCs and OA iPSCs were incubated in ODM to induce osteogenic differentiation (Figure 4). After 7 days of osteogenic induction, *in vitro* mineralization of cells was assessed by the OsteoImage Mineralization Assay Kit (Lonza). Fluorescent signals for mineralization were detected in RA iPSCs and OA iPSCs incubated with ODM, which means osteogenic cells were successfully differentiated from these iPSCs. This result shows that patient-derived iPSCs have a potential for bone formation.

## Discussion

Insights into the pathophysiology of RA have been derived largely from animal models such as collagen-induced arthritis, collagen antibody-induced arthritis, IL-1RaKO mice, and KBX/N serum transfer mice. However, the existing arthritic mouse model is not fully representative of the actual pathophysiology in patients with RA [16,17]. Indeed, it has been frequently suggested that insights from mouse models have rarely translated successfully to the clinic [18]. The production of simulation platforms and better access to genetically predisposed human cells may allow scientists to study the pathophysiology of diseases with greater ease. Previously, it was impossible to perform *in vitro* mechanistic studies and to produce *in vivo* transplant systems. The biological status obtained in simulation platforms using patient-derived iPSCs is now considered more representative of a disease than other systems. We thus generated human iPSCs from patients with RA (*n* = 2) and with OA (*n* = 2). Moreover, the osteogenic potential of RA iPSCs and OA iPSCs was shown by producing hydroxyapatite after stimulation with osteogenic differentiation. These results mean that patient-derived iPSCs have a potential of differentiation into tissues like other stem cells. We are going to investigate that these RA iPSCs and OA iPSCs could be suitable candidate materials for disease modeling and drug screening platforms.

The generation of disease-specific iPSCs has been attempted for many diseases [5,19-23]. The initial target diseases of iPSC generation were genetic deficiency diseases such as congenital anomalies or familial inherited diseases. Recently, iPSC studies have been extended to chronic diseases where the genetic etiology plays a less significant role than was the case in diseases studied previously. These diseases have mixed pathophysiological mechanisms with genetic background and environmental effects. The progression of RA is explained by a combination of susceptible genetic background and environmental exposure to *Porphyromonas gingivalis*, smoking, and other factors [24,25]. We thus consider that disease modeling may be applicable to RA after the generation of disease-specific iPSCs. Various environmental factors could be used to challenge an RA-simulated platform using iPSCs in a more sophisticated and individual-specific manner.

RA is characterized by its autoimmune mechanism, chronic inflammation, cartilage erosion, and bone destruction. In the final stage, the joints become dysfunctional. The destruction of cartilage and bone are the main causes of dysfunctionality and many scientists have tried to restore the defective areas. However, this approach has met with little success because of the cell sources used and immune rejection. Patient-specific iPSCs are regarded as good candidates for regeneration therapy because they may escape immunological rejection and exhibit pluripotency to differentiate into target cells such as cartilage and bone [13,26]. However, several issues need to be overcome before *in vivo* applications. Every material that is applied to humans needs to satisfy the rules of good clinical practice. In particular, good clinical practice guidelines prohibit the use of animal-derived products and oncogenic material, which is why the clinical application of iPSCs will require more time. Intensive efforts are ongoing to develop iPSCs using FBS-free media, feeder-free cultivation, and integration-free reprogramming, and numerous modifications are required to the protocol used for differentiation from iPSCs [27].

Two of the Yamanaka factors, *c-myc* and *klf4*, are regarded as oncogenes. Several modifications have been attempted to minimize the expression of these genes such as changing *c-myc* to *n-myc* and other combinations of factors. Interestingly, some somatic cells already expressed Klf4 and c-Myc, and therefore only required Sox2 and Oct4 for reprogramming [28]. In our experiments, RA and OA FLSs expressed Klf4 at a level that was suitable for establishing H7 stem cells. We thus expect that RA FLSs could be reprogrammed into iPSCs using minimized conditions, which would facilitate the minimal usage of oncogenes such as *c-myc* and *klf4*.

Occasionally, disease-specific iPSCs exhibit altered biological functions compared with ESCs, such as delayed cell proliferation and poor differentiation quality [2,3,29]. These phenomena were attributed to genuine problems with the primary cells that were reprogrammed. The disease state and drugs may thus affect the condition of primary cells. Our iPSCs had the full characteristics of stem cells according to immunostaining, karyotyping, and teratoma assays, but they exhibited slight alterations in their biological behavior. As various cytotoxic drugs such as methotrexate and other immunosuppressants are used in RA treatment, there is a significant possibility of prolonged exposure to cytotoxic drugs by RA FLSs, which could affect the reprogramming process. Further studies are needed to clarify the relationship between the quality of iPSCs and the primary somatic cells subjected to reprogramming.

## Conclusions

FLSs derived from RA and OA could be cell resources for iPSC reprogramming. Disease-specific and patient-specific iPSCs have the potential to be applied in clinical settings as source materials for molecular diagnosis and regenerative therapy. A molecular diagnostic approach, by disease in a dish using patient-specific iPSCs, may reveal complicated and individualized pathophysiology of RA. Regenerated tissue from patients’ own iPSCs will be a good material for tissue repair and structural restoration.

## Abbreviations

DMEM: Dulbecco’s modified Eagle’s medium; ESC: embryonic stem cell; FBS: fetal bovine serum; FLS: fibroblast-like synoviocyte; iPSC: induced pluripotent stem cell; OA: osteoarthritis; ODM: osteogenic differentiation medium; RA: rheumatoid arthritis; SCID: severe combined immune deficiency.

## Competing interest

The authors declare that they have no competing interests.

## Authors’ contributions

JL, YK, HY and JHJ carried out the molecular studies, cultured cells and drafted the manuscript. HJ carried out the immunohistochemical staining. JK and YAR performed the teratoma assay. MK and YGK participated in the karyotyping. JHJ, SMJ, S-HP and H-YK participated in the design of the study. JHJ and SD conceived of the study, and participated in its design and coordination. All authors read and approved the final manuscript.

## Authors’ information

JHJ is a visiting scholar at Stanford University and has participated in the project of reprogramming somatic cells to iPSCs. JHJ worked with JL and SD at the Stem Cell Institute of Stanford University. JHJ extended iPSC techniques to the rheumatologic field. Application of reprogramming techniques on RA FLSs to iPSCs was performed at The Catholic University of Korea. Karyotyping and teratoma formation were followed by the colleagues of Seoul St. Mary’s Hospital.

## Supplementary Material

Additional file 1 Is a table presenting the primer sequences used in the polymerase chain reaction. Click here for file

Additional file 2 **Is a figure showing positive expression of pluripotency markers on OA and RA iPSCs.** Other clones were generated during the reprogramming process. Immunofluorescence staining against Nanog, Oct4, Sox2, Tra-1-80, Tra-1-60, and SSEA-4 in RA and OA iPSCs. RA and OA iPSCs expressed high level of these markers. Click here for file
