# Supplementary figures and images for: Generation of disease-specific induced pluripotent stem cells from patients with rheumatoid arthritis and osteoarthritis
Source: Arthritis Res Ther. 2014 Feb 4;16(1):R41. doi: 10.1186/ar4470 (PMC3978583; doi:10.1186/ar4470)

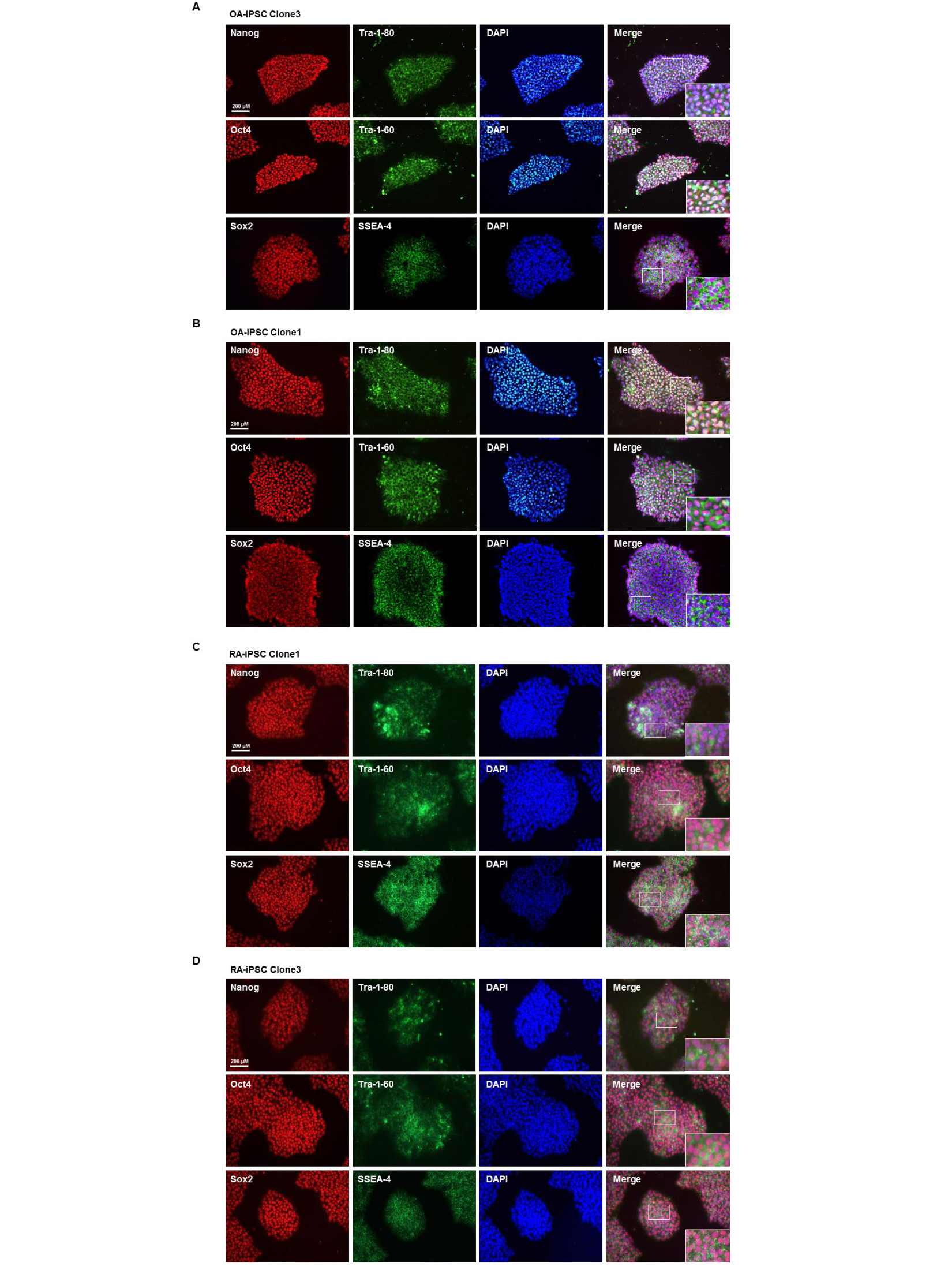

Supplement: Additional file 2 — Is a figure showing positive expression of pluripotency markers on OA and RA iPSCs. Other clones were generated during the reprogramming process. Immunofluorescence staining against Nanog, Oct4, Sox2, Tra-1-80, Tra-1-60, and SSEA-4 in RA and OA iPSCs. RA and OA iPSCs expressed high level of these markers. [file ar4470-S2.tiff]
